# Community Food Environment in Brazilian Medium-Sized Municipality After the Ore Dam Break: Database Creation and Diagnosis

**DOI:** 10.3390/ijerph22111723

**Published:** 2025-11-14

**Authors:** Patrícia Pinheiro de Freitas, Mariana Souza Lopes, Nathália Luíza Ferreira, Sérgio Viana Peixoto, Aline Cristine Souza Lopes

**Affiliations:** 1 Grupo de Pesquisa de Intervenções em Nutrição (GIN/UFMG), Universidade Federal dos Vales do Jequitinhonha e Mucuri, Diamantina 39100-000, Minas Gerais, Brazil; patricia.freitas@ufvjm.edu.br; 2 Grupo de Pesquisa de Intervenções em Nutrição (GIN/UFMG), Universidade Federal da Paraíba, João Pessoa 58051-900, Paraíba, Brazil; marianalopes.ufpb@gmail.com; 3 Núcleo de Estudos e Pesquisas em Epidemiologia e Nutrição (NEPEN), Grupo de Pesquisa de Intervenções em Nutrição (GIN/UFMG), Universidade Federal de Lavras, Lavras 37200-900, Minas Gerais, Brazil; nathalia.luiza@ufla.br; 4 Departamento de Gestão em Saúde, Universidade Federal de Minas Gerais, Belo Horizonte 31270-901, Minas Gerais, Brazil; sergio.peixoto@fiocruz.br; 5 Núcleo de Estudos em Saúde Pública e Envelhecimento, Instituto René Rachou, Fiocruz Minas, Belo Horizonte 30190-009, Minas Gerais, Brazil; 6 Grupo de Pesquisa de Intervenções em Nutrição (GIN/UFMG), Universidade Federal de Minas Gerais, Belo Horizonte 30130-100, Minas Gerais, Brazil

**Keywords:** disasters, food environment, food retail, validation studies

## Abstract

This study proposed a methodology for obtaining a valid database of food retail establishments and characterized the community food environment, understood as the distribution and type of food outlets, in a Brazilian medium-sized municipality after the collapse of a mining tailings dam. An ecological study was conducted with establishments selling food for home consumption (butcher shops, fish markets; fruit and vegetable specialty markets; large- and small-chain supermarkets; bakeries and local markets) and immediate consumption (bars, snack bars, and restaurants). For home-consumption establishments, data were requested from governments and completed with website/app searches, virtual audits (Google Street View), and on-site audits. For immediate-consumption establishments, only on-site audit was used due to the low quality of the secondary databases. Agreement between databases was assessed with the Kappa statistic. Density (d) was calculated by the area (in km^2^) of the sampling stratum. Public databases presented low validity (23.0%; Kappa −0.388; *p* = 1.000), even after virtual auditing (31.4%; Kappa 0.37; *p* < 0.001). 96 establishments for home consumption and 261 for immediate consumption were identified, with predominance of local markets (35.4%), bars (35.2%), and snack bars (29.1%). The region with the highest density of establishments was the “Other Areas” stratum (d = 4.7 for home-consumption establishments and d = 13.2 for immediate-consumption establishments). Audit proved most effective, especially for small establishments. The lack of governmental databases and the identified food environment should inform municipal policies to promote food and nutrition security and reduce inequalities after the disaster.

## 1. Introduction

The built environment is a multidimensional concept defined by the physical, economic, political, and sociocultural aspects of the places where individuals live, study, work, and commute [1,2]. When it incorporates elements such as the availability, accessibility, and convenience of food, it is referred to as the food environment. The food environment is, therefore, the connection between the food system and the consumer, influencing food choices and nutritional status [1,2,3,4].

The food environment has drawn increasing scientific interest, with a substantial rise in studies investigating its association with health outcomes [5,6,7,8]. However, its assessment faces methodological challenges related to the sources used to obtain data on food retail establishments and their validity, especially in small- and medium-sized municipalities [6,9].

The most common methods for obtaining information on the food environment are on-site audits and the use of secondary databases provided by governmental or private organizations. The first method is considered the gold standard and involves direct observation of food retail establishments, but it entails high costs and requires substantial logistics and time for execution [7,10]. The second method, while subject to outdated databases that may produce inaccurate results, is highly feasible due to its practicality and lower cost [7]. To address the limitations of using secondary databases, virtual audits via Google Street View^®^ have been employed [6]. Nevertheless, limitations persist, such as photo coverage being restricted to areas where Google^®^ imagery is available, image quality hindering verification, or storefronts not allowing adequate characterization of establishments. Furthermore, images may be unavailable at the request of owners or may predate the opening of the establishment [6].

In small- and medium-sized municipalities, where administrative structures may be less digitized or systematized, public databases may be even more outdated, resulting in the omission of food retail establishments. Moreover, updates to Google^®^ databases may occur less frequently than in major metropolitan areas. These information biases tend to be exacerbated in contexts of institutional instability, as is the case in Brumadinho in Minas Gerais, the municipality under study.

In 2019, Brumadinho experienced a disaster involving the collapse of a mining tailings containment dam. Dam failures may not only cause immediate physical and psychological trauma but may also disrupt social and environmental determinants of health, such as access to clean water, adequate food, and safe housing conditions. Contamination of soil and water used in agriculture and fishing may have compromised the quality and availability of food, especially that from local production [11,12]. As a consequence, this disaster could have affected the food supply of communities and access to water, compromising Food and Nutritional Security, altering dietary patterns, and increasing vulnerability to health problems. The disaster could also have affected local commerce and the municipality’s capacity to maintain and update information systems, such as revenue records. Thus, the effects of the disaster are not limited to immediate damage but rather constitute far-reaching public health emergencies that can also compromise the food environment [13].

Given the multiple limitations of food environment data sources, obtaining a reliable database of food retail establishments may require combining different data sources [7]. In this context, this study aimed to propose a methodology to obtain a valid database of food retail establishments in Brumadinho, Minas Gerais, and to characterize the community food environment, that is, the distribution and typology of food outlets after the disaster that occurred in the municipality. This research advances current methodologies for assessing food environments by adapting and validating procedures specifically for medium-sized municipalities, a setting that remains underrepresented in the scientific literature. In addition, this study examined the food environment in a post-disaster, which is unprecedented in the literature. The validated database developed in this work can serve as a foundation for future longitudinal and inferential studies. By generating reliable data on the spatial and structural dimensions of the local food environment, this study provides evidence to inform municipal actions that can promote food and nutrition security and reduce inequalities in access to healthy foods. The database construction process used in this research can also serve as a basis for future studies in a similar context.

## 2. Methods

### 2.1. Study Design and Setting

This ecological study is part of the *Projeto Saúde Brumadinho* (Brumadinho Health Project), conducted in Brumadinho, Minas Gerais, Brazil, which aims to produce longitudinal information on the health of the population residing in the municipality after the collapse of a mining tailings dam in 2019 [14]. For this purpose, the study investigated areas directly affected by the dam collapse, areas with ongoing mining activity, and areas not directly exposed to tailings mud or mining operations. This specific analysis focused on the investigation of the food environment and is part of the fourth wave of data collection from the Brumadinho Health Project, conducted in 2024.

Brumadinho has a high Municipal Human Development Index (MHDI) (0.747) and a population of 38,915 inhabitants distributed over 639.434 km^2^, according to the 2022 Population Census. Mining constitutes the primary economic activity of the municipality. Nevertheless, agriculture remains a significant source of employment and income, mainly through the production of fruits, vegetables, and other horticultural goods by smallholder and family farmers. Furthermore, the municipality has shown steady growth in tourism-related activities in recent years [15]. In 2019, the collapse of the Córrego do Feijão mine tailings dam, operated by Vale S.A., affected 297.3 hectares of municipal land and caused approximately 270 deaths [13]. The collapse of the mining tailings containment dam may have caused immediate food insecurity and affected access to clean water, adequate food, and safe housing conditions. In addition, contamination of soil and water used in agriculture and fishing may have compromised the quality and availability of food, especially that from local production. In this way, Brumadinho was selected as the study site because the scale and severity of this disaster provide a unique context to examine the environmental, economic and health-related changes in food access and availability in this setting, which is essential to understand how environmental disasters can disrupt local and food systems and influence population health [1,4].

### 2.2. Data Collection

All stages of the methodology for constructing a valid database were carried out by a team of nutritionists and undergraduate Nutrition students, supervised by a field coordinator. The entire team was previously trained using a field manual developed explicitly for data collection.

The virtual audit was conducted individually under supervision. The on-site audit, also supervised, was conducted in pairs between 29 July and 16 August 2024, based on an address list of food retail establishments and maps generated using Google My Maps^®^. The researchers monitored all data collection activities through daily field reports.

### 2.3. Database Construction

#### 2.3.1. Food Retail Establishments for Home Consumption

Establishments eligible for the study included all those selling food for home consumption or immediate consumption, according to the criteria of the *Classificação Nacional de Atividades Econômicas* (CNAE, as in Portuguese—Brazilian National Classification of Economic Activities) (Supplementary Material/Table S1), located within the census tracts of the three sampling domains of the Brumadinho Health Project: areas directly affected by the dam collapse, areas with mining activity, and areas not directly exposed to tailings mud or mining operations.

For home consumption, the following types of establishments were included: butcher shops; fish markets; fruit and vegetable (FV) specialized market; hypermarkets; supermarkets; bakeries/confectioneries; dairy and cold cuts retailers; candy, sweets, and chocolate retailers; and minimarkets/grocery stores/warehouses. For immediate consumption, the following were included: restaurants (à la carte, self-service, rodízio, per kilogram, fast-food style, or snack bars), bars, ice cream shops, delivery services, bakeries, and coffee shops. Establishments were excluded if located in rural areas, closed during data collection, operating exclusively for parties or events, or unclassifiable by type.

**Figure 1:**
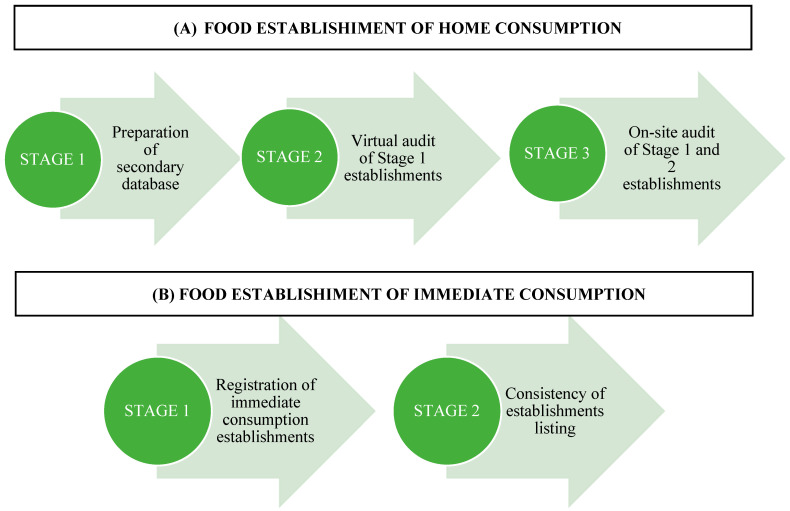
Stages involved in creating a database of food environments databases in Brumadinho, Minas Gerais, 2024.

The database for home consumption establishments was built in three stages (Figure 1). First, secondary databases were requested from the State Secretariat of Finance of the Government of Minas Gerais and the Municipal Secretariat of Finance of Brumadinho; for street markets, the Municipal Secretariat of Agriculture was contacted. The State Secretariat reported that establishment records were kept only at the municipal level. The municipality, however, reported having no listing of food retail establishments and no street markets under municipal coordination due to low attractiveness for producers.

Given the unavailability of governmental secondary databases, the research team compiled a database using information from the following websites and apps (Figure 1): (1) Open Data Portal (dados.gov.br); (2) “Empresa Aqui” website [https://www.empresaqui.com.br/listas-empresas?gad_source=1&gad_campaigned=18665571724&gbraid=0AAAAABZVDXucB-dX3qbJb4bXjg70Inuxm&gclid=CjwKCAiAoNbIBhB5EiwAZFbYGPMsC-l8OJQaR--rultzhA4s04xpYvL6LhBrJr7EEEfP7SGX6HojoBoCiBcQAvD_BwE] (Accessed on 2 May 2024); (3) “Lista de Empresas” [https://www.listasdeempresa.com/] (Accessed on 2 May 2024); (4) Brumadinho commercial directory [https://brumadinho.portaldacidade.com/guia-comercial] (Accessed on 2 May 2024); (5) direct searches on Google^®^; and (6) establishments registered on the IFood^®^ app.

From the private secondary databases, establishments for home consumption were selected and classified according to the sales characteristics described by CNAE (Supplementary Material/Table S1). A single database was then compiled, with duplicate establishments removed.

The second stage consisted of a virtual audit using Google Street View^®^, following the non-commercial use guidelines and Google Terms of Service [https://www.google.com/permissions/geoguidelines/; https://policies.google.com/privacy?hl=en-US] (Accessed on 15 May 2024); [https://www.google.com/intl/pt_US/help/terms_maps/] (Accessed on 15 May 2024) (Figure 1). Establishments located inside enclosed commercial centers were verified based on their presence in the official store directories of those locations.

The third stage involved on-site audits for direct observation of establishments. Before the data collection phase began, the field supervisor created maps showing the routes that the team should follow. These routes were designed to cover all commercial establishments and streets within the surveyed census tracts. The maps were shared with the research team via ‘My Maps’ and also provided in printed form to allow consultation in locations without internet access. The research team also received a spreadsheet containing the commercial name and address of each establishment. This spreadsheet was used to record the data collected, or to note reasons for not collecting data (e.g., the establishment does not exist or is closed). If the research team encountered other establishments not listed on the route, these were to be included in the list, with data to be collected from them (Figure 1).

#### 2.3.2. Food Retail Establishments for Immediate Consumption

Given the low consistency of secondary data and the limitations observed in the virtual audit establishments for home consumption, we chose not to include this step in the investigation of immediate-consumption establishments. So, the first stage in building the immediate consumption database consisted of recording establishments encountered along the audit routes for home consumption establishments. Data were collected using a Google Forms^®^ instrument to record the trade name and address (Figure 1).

The second stage involved reviewing the list obtained and removing duplicates after checking names and addresses. Immediate consumption establishments were classified according to type, based on CNAE criteria (Supplementary Material/Table S1). Mixed establishments were defined as those combining characteristics of more than one type (e.g., restaurant and bar, or restaurant and snack bar), with different activities possibly occurring at different times of day.

### 2.4. Data Analysis

The proportion of home consumption establishments with discrepancies between the secondary database, the virtual audit (% agreement in the virtual audit), and the on-site audit (% agreement in the on-site audit) was calculated.

The percentage agreement between virtual and on-site audits was estimated using the Kappa statistic (κ), which measures the level of agreement beyond chance. It is calculated as κ = (Po − Pe)/(1 − Pe), where Po represents the observed agreement and Pe the expected agreement by chance. Kappa values range from −1 to 1, with higher values indicating stronger agreement, according to Landis and Koch’s classification [16]: ≤0 no agreement, 0.01–0.20 slight, 0.21–0.40 fair, 0.41–0.60 moderate, 0.61–0.80 substantial, and 0.81–1.00 almost perfect agreement.

After the on-site audit, all establishments—both for home and immediate consumption, as well as newly identified ones—were georeferenced using Google Maps^®^. Latitude and longitude in decimal degrees were converted to UTM coordinates using the automatic converter available at https://splink.cria.org.br/conversor?criaLANG=en (Accessed on 10 June 2024) and, when necessary, the geographic calculator of DPI/INPE [http://www.dpi.inpe.br/calcula/] (Accessed on 10 June 2024).

From the validated database, a descriptive analysis of the community food environment was carried out according to the sampling domains of the Brumadinho Health Project: (1) Impacted Area—census tracts directly affected by the dam collapse; (2) Mining Area—census tracts with mining activity; and (3) Other Areas—census tracts not directly exposed to tailings mud or mining operations. Mixed census tracts initially classified as “Other Areas” but inhabited by individuals recognized as belonging to impacted or mining areas were also identified. This reclassification resulted from discussions with community leaders and study participants, considering territorialization as a dynamic process shaped by residents’ perceptions. Two new mixed categories were thus defined: (4) Mining and Other Areas; and (5) Impacted and Other Areas.

Additionally, thematic maps were created, stratified by establishments for home and immediate consumption. The density of establishments for home and immediate consumption was calculated by dividing the number of establishments identified by the area (in km^2^) of each sampling stratum. Data organization and spatialization were performed using QGIS 3.40.2 [17], and statistical analyses were conducted in Stata version 14. A significance level of 5% was adopted for all analyses.

## 3. Results

**Figure 2:**
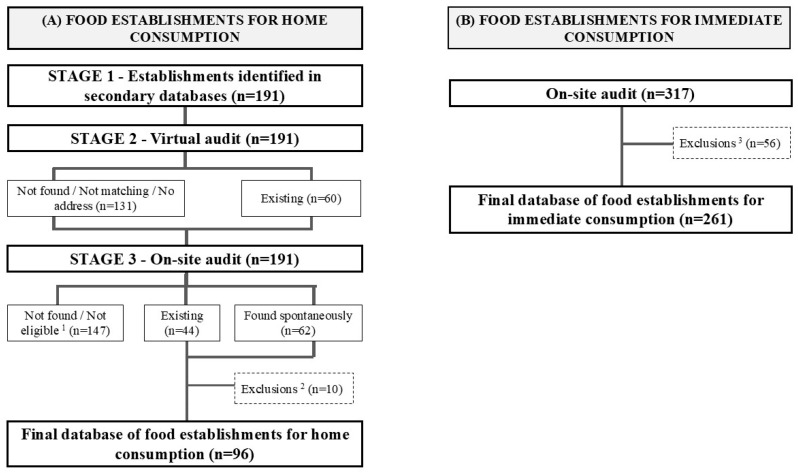
Results of the stages involved in creating a database of food establishments in Brumadinho, Minas Gerais, Brazil, 2024. ^1^ Not eligible: Commercial establishment for immediate consumption (n = 8), Non-existent/Address not found (n = 126), Closed/Does not correspond to the listed establishment (n = 11), Rural area (n = 2); ^2^ Closed commercial establishments (n = 5); Establishments in census tracts without study participants (n = 5); ^3^ Excluded: Repeated establishments (n = 20); Not eligible [permanently closed (n = 8); Non-existent (n = 8); Operating only for events or with reservations (n = 2); Home consumption establishments (n = 6); Establishments in census tracts without study participants (n = 12)].

The secondary database of food establishments for home consumption built in Stage 1 included 191 establishments eligible for virtual auditing in Stage 2. Due to the low number of establishments found in the virtual audit (n = 60), the decision was made to proceed with on-site auditing (Stage 3) for all 191 establishments (Figure 2). Sixty food retail establishments for home consumption were concordant between the secondary database and the virtual audit (31.4%), and 44 establishments were concordant between the secondary database and the on-site audit (23.0%). In the on-site audit, 62 new establishments were found, of which five were excluded because they were closed and another five because they were located in census tracts without study participants, resulting in a final database with 96 food retail establishments for home consumption representing a 49.7% reduction from the database proposed in Stage 1 after the on-site audit (Figure 2).

When calculating the degree of agreement beyond chance (Kappa index) between databases obtained by different methods, variations in the consistency of results were observed. The comparison between the virtual audit and the on-site audit showed a Kappa index of 0.37 (*p* < 0.001), indicating fair, statistically significant agreement. In contrast, the comparison between the secondary database and the on-site audit showed a negative Kappa index (−0.388; *p* = 1.000), suggesting that the two methods exhibit a pattern of divergence greater than would be expected by chance.

For immediate consumption establishments, 317 establishments were found during the on-site audit. After excluding ineligible establishments (n = 24) and duplicates (n = 20), the final database included 261 establishments (Figure 2).

**Figure 3:**
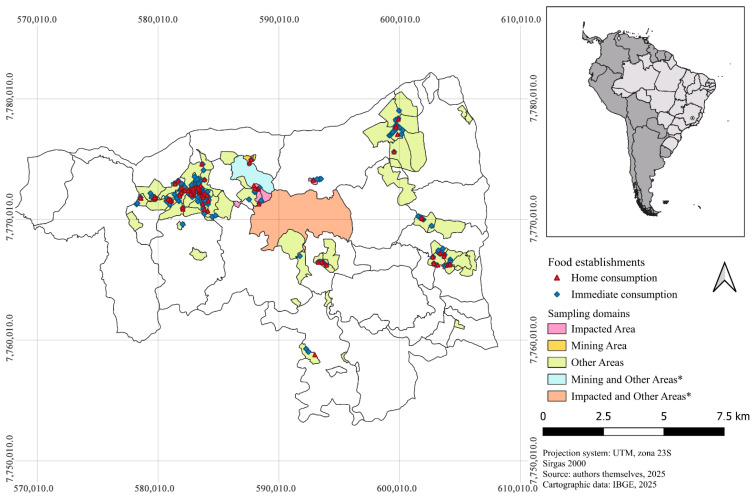
Spatial distribution of food establishments according to sampling strata in Brumadinho, Minas Gerais, Brazil, 2024. * Census sectors in which participants were classified into different research domains.

Figure 3 shows the spatial distribution of commercial establishments selling food for home and immediate consumption in the municipality of Brumadinho-MG, according to the sampling strata. A trend toward a higher concentration of food retail establishments was identified in the western geographic region of Brumadinho, in the central area of the municipality, where the main commercial and residential areas are located. Small clusters of establishments were observed in regions of settlements and residential neighborhoods. Areas classified as the “Mining Area” and the “Impacted Area” presented lower number of commercial establishments (n = 21). In the “Mining Area,” 2 establishments for home consumption (density = 0.41 establishments/km^2^) and 4 for immediate consumption (density = 0.64 establishments/km^2^) were identified. In “Impacted Area,” 7 establishments for home consumption (density = 2.86 establishments/km^2^) and 8 for immediate consumption (density = 1.75 establishments/km^2^) were found. In “Other Areas,” there was a marked concentration of food retail establishments, with 87 for home consumption (density = 4.70 establishments/km^2^) and 249 for immediate consumption (density = 13.22 establishments/km^2^).

**Figure 4:**
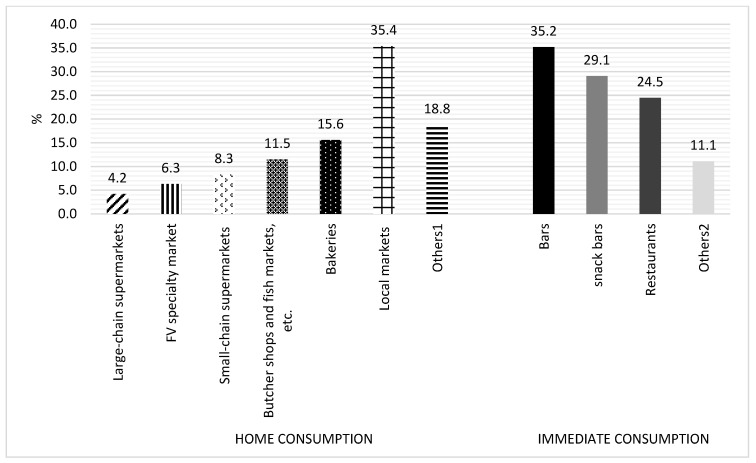
Distribution of types of food establishments according to type in Brumadinho, Minas Gerais, Brazil. 2024. ^1^ Others: Mixed establishments (n = 3); Deliveries (n = 3). ^2^ Others: Distributors (n = 5); Emporiums (n = 4); Drugstores (n = 3); Convenience and candy stores (n = 2).

The prevalence of food retail establishments by type, stratified by home versus immediate consumption, can be seen in Figure 4. Among establishments for home consumption, local markets were the most prevalent (35.4%), followed by bakeries (15.6%), butcher shops and fish markets (11.5%), small-chain supermarkets (8.3%), FV specialty market (6.3%), and large-chain supermarkets (4.2%). Among immediate consumption establishments, 35.2% were bars, 29.1% snack bars, and 24.5% restaurants.

## 4. Discussion

This study revealed important limitations in using public databases to characterize the food environment from a medium-sized municipality—Brumadinho, Minas Gerais— stemming from the unavailability of governmental sources and the low validity of information obtained from websites. Thus, on-site auditing proved to be the appropriate strategy for assessing the municipality’s food environment. In the post-mining dam collapse scenario, small businesses predominated, especially those catering for immediate consumption, such as bars and snack bars. These businesses were concentrated in the central region, where residences, services and commerce are concentrated. This area was not directly affected by the disaster or by mining activity.

In the absence of governmental sources, a methodology was proposed to build a valid database to investigate the food environment in Brumadinho, MG. Public databases accessed via websites and apps, and virtual audits were not helpful, as nearly half of the establishments could not be confirmed in the on-site audit. The on-site audit stood out as the method with the highest validity for the context investigated. However, it is worth noting that analyzing the food environment using this method was only possible in Brumadinho because it is a medium-sized municipality, and because the necessary financial and human resources for data collection were available. These conditions are not always present in other studies, which reiterates the importance of using updated and valid secondary sources to enable research across diverse contexts [18].

Secondary data sources are highly relevant for food environment research because they allow rapid, lower-cost, large-scale investigations. A systematic review on the validity of secondary databases identified high variability in agreement between secondary data and on-site auditing, with the type of secondary source being an important determinant of this variability [19]. Studies conducted in Cambridgeshire County, United Kingdom [20], and in Newfoundland and Labrador, Canada [21] reported overall agreement between secondary databases and on-site auditing of 49% and between 17% and 45%, respectively. Similarly, a study conducted in Belo Horizonte, the capital of Minas Gerais State, reported 45.7% agreement [22].

Consistent with the literature, our results indicated the low relevance of secondary databases, as evidenced by the negative agreement found. Among the different factors, the lower sensitivity of secondary databases may be explained by the existence of informal establishments and by the dynamic opening and closing of commercial establishments, making any static description of the food environment imprecise over time [18,19]. In Brumadinho, however, the absence of a government database, whether local or state, may have significant consequences for local management, including reduced tax revenue and inadequate sanitary inspections [19]. Moreover, control and monitoring of the food retail sector are essential for planning and developing public policies on food supply and the promotion of adequate and healthy eating to ensure the population’s health and food and nutrition security (SAN), especially after a disaster such as the one experienced in Brumadinho.

The spatial distribution of establishments for home and immediate consumption showed similar characteristics, being located predominantly in the central region of the municipality. Conversely, the low number of establishments found in mining areas and areas impacted by the dam collapse may be a consequence of the destruction caused by the disaster, as well as the harms associated with mining activity. This concentration of commercial establishments may limit food access for populations living in peripheral areas. Different studies have shown that regions with lower concentrations of food retail establishments are also areas where people with worse socioeconomic conditions, who self-identify as Black and/or Brown and are linked to cash transfer programs, predominantly reside [8,23,24], contributing to the perpetuation of inequalities in access to food and services, with repercussions for the realization of the rights to health and adequate food. Nevertheless, it is necessary to consider the dynamic nature of territorial occupation in cities. The concentration of food establishments in a given region is typical of municipalities with large geographic areas but with population concentrated in a specific area, as in Brumadinho [25]. The most recent Census data available for Brumadinho (2010) showed that 84.3% of the population resided in urban areas, driven by the development of economic and tourist activities [25].

In the municipality, local markets were predominant for home consumption. This scenario reflects a pattern distinct from large Brazilian metropolises, where larger supermarkets and hypermarkets predominate [26,27,28]. The small establishments identified are characterized by counter service, in which an employee serves the customer, often a family member who retrieves the goods and frequently conducts trust-based sales, recorded in a ledger (“caderneta”) [29]. This retail format is increasingly rare in the country. However, it is still observed in smaller municipalities and in neighborhoods where merchants and residents know one another and establish relationships of trust. However, this type of establishment tends to offer low diversity, variety, and quality of fruits and vegetables, along with a high supply of ultra-processed foods with strong advertising appeal [30], contributing to obesogenic food environments [1,4]. In a post-disaster setting such as Brumadinho, this limited access to fresh and minimally processed foods may exacerbate food insecurity and nutritional vulnerability [31,32], particularly among populations already facing social and economic instability [33,34,35]. Over time, these conditions can contribute to unhealthy dietary patterns characterized by high consumption of ultra-processed products, increasing the risk of overweight, obesity, and diet-related non-communicable diseases [1,4].

Concerning food retail for immediate consumption, establishments considered unhealthy such as bars and restaurants predominated. Research conducted in other medium-sized municipalities in Minas Gerais showed similar characteristics. In Ouro Preto, a higher concentration of unhealthy establishments (convenience stores, small groceries, bars, candy shops, snack bars, and ice cream shops) was also observed in central and wealthier areas. In contrast, few establishments were located in lower-income areas [8]. In Lavras, there was a predominance of mixed establishments (restaurants, bakeries/confectioneries, dairy shops, minimarkets/grocery stores/warehouses, supermarkets/hypermarkets, mobile food services, among others) [23].

Bars and restaurants also predominate in investigations of the food environment in large municipalities [26,36,37,38]. These establishments focus on the sale of alcoholic beverages and snacks and may also offer a variety of ultra-processed foods, especially sugar-sweetened beverages and sweets. They employ sales strategies that encourage the consumption of ultra-processed foods, such as visual advertisements for alcoholic beverages, “combo” deals that include sugar-sweetened beverages with snacks, and discounts for upsizing soft drink portions [39]. In addition, the availability of healthy food options is low and, when available, they are more expensive than ultra-processed foods [39], which may favor excessive consumption of ultra-processed foods, whose health impacts are well known [4]. In this context, the predominance of bars, snack bars, and other immediate-consumption outlets reinforces an environment that promotes the intake of high-calorie, nutrient-poor foods, which may further compromise diet quality and aggravate the long-term burden of non-communicable diseases in communities recovering from environmental disasters. 

In addition to this, there are results from a previous study conducted in Brumadinho, MG, showed that less than half of the population had regular or recommended consumption of unprocessed and minimally processed foods, with an association with the impact of area of residence due to the dam collapse [40]. In parallel, 35.1% of families experienced food insecurity, reporting reduced income following the dam collapse. These results highlight the vulnerability of families in ensuring the right to adequate food [41].

This study has limitations. The secondary database was not used to obtain information on immediate consumption establishments, which prevents drawing inferences about their validity. However, since the databases for home consumption establishments were not valid, a similar situation may occur for immediate consumption establishments. So, given the divergences observed between commercial activities identified in the on-site audit and governmental databases, this is believed to have been an appropriate decision.

It was not possible to infer the consequences of the disaster for the municipality’s food environment because no prior assessment was available. However, the results will allow future longitudinal comparisons, making it possible to verify whether the concentration of economic activity related to food commerce in the central region persists, in contrast to mining areas and areas impacted by the disaster.

The study also did not include population density data to make inferences about food access, nor information on establishment characteristics such as variety, diversity, advertising, and food prices, which affect dietary intake. It is worth noting, however, that evaluating the consumer food environment was not the objective of this study. Using the conceptual framework proposed by Glanz et al. [2], this research focused on the community food environment, which refers to the distribution, density, and type of food retail establishments within a given geographic area. This dimension differs from the consumer food environment, which encompasses the internal characteristics of establishments—such as price, promotion, and availability—that influence purchasing decisions. By prioritizing the community dimension, the present study contributes to understanding how the spatial organization and typology of food outlets shape access to food and may reinforce or mitigate social inequalities, particularly in post-disaster contexts. Such evidence is fundamental to informing municipal policies aimed at promoting food and nutrition security, guiding strategic interventions to expand the availability of unprocessed and minimally processed foods and to strengthen local commerce in vulnerable territories.

In addition, because establishment types were determined by on-site auditing, their characteristics were defined by the research team rather than by consulting the official CNAE registry; this strategy was adopted due to the absence of governmental databases. It should be emphasized, however, that the research team was trained to identify establishments based on each CNAE description [19].

This study presents several methodological strengths that advance the field of food environment research, particularly in post-disaster contexts. The rigorous, multi-method validation process and the site audits ensured the creation of a reliable and high-quality georeferenced dataset of food retail establishments. This approach not only allowed for critical assessment of the accuracy and completeness of secondary data sources but also provided a validated database that can serve as a foundation for future longitudinal and comparative studies. Furthermore, by demonstrating the feasibility of this methodological framework in a medium-sized municipality affected by a large-scale environmental disaster, this work fills a significant gap in the literature and offers a replicable model for similar contexts.

This work has implications for research and public health policy, and it also aligns with mission to advance the knowledge of the environmental determinants of health. The results emphasize the importance of having secondary databases available and assessing their validity; they reflect on the difficulties of obtaining such databases in medium-sized municipalities; and they suggest that, to ensure adequate data quality on the food environment in these municipalities, on-site auditing is the best strategy, corroborating the results of others [19,20,21,22]. Furthermore, these findings can inform practical actions at multiple levels of governance. At the local level, municipal health, food and nutritional security and agriculture departments can adopt standardized on-site audit tools to continuously monitor food availability and access. At the state and federal levels, agencies can invest in building and maintaining validated food environment databases that integrate geographic, health, and environmental data aiming to map uncovered areas of establishments that sell healthy foods. This is particularly important in small and medium-sized municipalities, which face greater challenges in keeping their databases up to date. Such coordinated strategies can enhance preparedness and response capacity in the event of future environmental disasters, ensuring that food systems and population health surveillance remain robust and evidence-based.

In this regard, the study fills a knowledge gap on the validity of secondary databases in medium-sized municipalities, presenting novel results on the community food environment after a human-caused disaster. A spatial concentration of establishments for home and immediate consumption was observed in the central region, which may limit access in more peripheral regions. Moreover, the predominance of local markets for home consumption and of bars and snack bars for immediate consumption may compromise the healthfulness of available foods.

## 5. Conclusions

In Brumadinho, Minas Gerais, on-site auditing proved to be the most effective strategy to obtain accurate data on food retail establishments. The study identified a predominance of small food establishments, particularly those for immediate consumption, and a spatial concentration in central areas. These findings highlight the need for local policies that stimulate the commercialization of unprocessed and minimally processed foods to promote population health and reduce inequalities in access to food. In addition, greater oversight of reliable municipal databases by governmental organizations is required to support economic planning, sanitary surveillance, and intersectoral public policies.

## Data Availability

The raw data supporting the conclusions of this article will be made available by the authors on request.

## Supplementary Materials

The following supporting information can be downloaded at: https://www.mdpi.com/article/10.3390/ijerph22111723/s1. Table S1: Food Retail Establishment Types according CNAE.
