# Supplementary material for: Community Food Environment in Brazilian Medium-Sized Municipality After the Ore Dam Break: Database Creation and Diagnosis
Source: Int J Environ Res Public Health. 2025 Nov 14;22(11):1723. doi: 10.3390/ijerph22111723 (PMC12652863; doi:10.3390/ijerph22111723)
Supplement: Supplementary file 1 [file ijerph-22-01723-s001.zip › ijerph-3899533-supplementary.pdf]

Supplementary Material 1 – Food Retail Establishment Types according CNAE

**Butcher shop / fish markets / meat market**	Retail trade specializing in the sale of beef, pork, goat, sheep, and equine meat, either fresh, chilled, or frozen
**Municipal fruit and vegetable market**	Retail outlet primarily selling fruits, vegetables, and legumes, although other products, such as canned goods, may also be available.
**Fruit and vegetable (FV) specialized market**	Permanent retail establishment specializing in the sale of fresh produce. These are typically small outlets supplied according to the owner's purchasing arrangements.
**Minimarkets/grocery stores/warehouses**	Retail stores selling goods over the counter, where customers are served by an employee who retrieves the items. The traditional "trust-based" system of recording purchases in notebooks is still practiced, although checks and credit cards are also accepted. Employees are usually family members.
**Supermarkets**	Retail outlets offering medium price levels, limited autonomy over pricing and purchasing policies, and standardized architectural design according to the area profile. Food items—especially perishables—predominate in the product mix.
**Hypermarkets**	Retail stores located in large, high-traffic areas with easy access, intended for bulk purchases. They offer lower prices and greater discounts. Their architectural design is simpler, and non-food items account for a larger share of the product mix, including complementary goods and services such as household and automotive accessories.
**Wholesale stores ("Atacadistas")**	Large retail-like outlets selling products individually, similar to regular retail stores.
**Bakeries / confectioneries**	Businesses engaged in the production and/or sale of bakery items such as bread, rolls, cakes, pies, dairy products, cold cuts, sweets, savory snacks, sandwiches, and ice cream.
**Others**	Delicatessens, specialty shops (dairy and cold cuts retailers, candy, sweets, and chocolate retailers), pharmacies, convenience stores, and similar establishments.
